# Predictors of Colon Cancer Screening Among the Saudi Population at Primary Healthcare Settings in Riyadh

**DOI:** 10.3390/curroncol32050243

**Published:** 2025-04-22

**Authors:** Amani Alharthy, Mamdouh M. Shubair, Badr F. Al-Khateeb, Lubna Alnaim, Emad Aljohani, Nada Kareem Alenazi, Maha Alamodi Alghamdi, Khadijah Angawi, Rawabi M. Alsayer, Naif M. Alhawiti, Ashraf El-Metwally

**Affiliations:** 1 Department of Health Science, College of Health and Rehabilitation Sciences, Princess Nourah Bint Abdulrahman University, Riyadh 13412, Saudi Arabia; afalharthy@pnu.edu.sa; 2 School of Health Sciences, Faculty of Human and Health Sciences (FHHS), University of Northern British Columbia (UNBC), 3333 University Way, Prince George, BC V2N 4Z9, Canada; 3 King Abdullah International Medical Research Center, Riyadh 11481, Saudi Arabia; khateebb@ngha.med.sa (B.F.A.-K.); hawitin@ksau-hs.edu.sa (N.M.A.); elmetwallya@ksau-hs.edu.sa (A.E.-M.); 4 Department of Epidemiology and Biostatistics, College of Public Health and Health Informatics, King Saud Bin Abdulaziz University for Health Sciences, Riyadh 14611, Saudi Arabia; 5 King Abdulaziz Medical City, Ministry of National Guard—Health Affairs, Riyadh 11426, Saudi Arabia; 6 Clinical Nutrition Department, College of Applied Medical Sciences, King Saud Bin Abdulaziz University for Health Sciences, Al Ahsa 36428, Saudi Arabia; nuaimlu@ksau-hs.edu.sa; 7 Department of Surgery, College of Medicine, Prince Sattam Bin Abdulaziz University, Al-kharj 16278, Saudi Arabia; e.aljohani@psau.edu.sa; 8 Ministry of Health, Riyadh Second Health Cluster, Riyadh 12382, Saudi Arabia; nkalenazi@moh.gov.sa; 9 King Fahad Medical City, Riyadh 12231, Saudi Arabia; 10 Department of Surgery, College of Medicine, King Khalid University, Abha 62521, Saudi Arabia; malamodi@kku.edu.sa; 11 Department of Health Services and Hospital Administration, Faculty of Economics and Administration, King Abdulaziz University, Jeddah 22254, Saudi Arabia; kkangawi@kau.edu.sa; 12 Population, Public & Environmental Health, Health Research Center, Ministry of Defense Health Services General Directorate, Riyadh 12426, Saudi Arabia; rralsayer@gmail.com; 13 Department of Clinical Laboratory Sciences, College of Applied Medical Sciences, King Saud Bin Abdulaziz University for Health Sciences, Riyadh 11481, Saudi Arabia

**Keywords:** predictors, colorectal cancer, screening, Saudi Arabia

## Abstract

(1) Background: This study aims to identify the sociodemographic, behavioural, and systemic predictors of colorectal cancer (CRC) screening among primary healthcare attendees in Riyadh, Saudi Arabia, to inform targeted interventions and policy strategies. (2) Methods: This cross-sectional study was conducted between March and July 2023 across 48 randomly selected primary healthcare centers in Riyadh, Saudi Arabia. The target population for this study was adults aged 18 and above attending primary healthcare centers in Riyadh. Multi-stage random sampling was used to recruit participants. Multivariate logistic regression was performed to identify independent predictors of CRC screening. (3) Results: CRC screening uptake was found to be only 4.2%. Age was a significant predictor, with individuals aged 50–75 years (adjusted odds ratio [AOR]: 1.90, 95% confidence interval [CI]: 1.50–2.42) and those aged 75 years or older (AOR: 1.37, 95% CI: 1.01–1.87) being more likely to undergo screening compared to younger individuals. Insurance coverage strongly influenced screening behaviour (AOR: 1.64, 95% CI: 1.37–1.96). Smokers were nearly four times more likely to participate in screening than non-smokers (AOR: 3.87, 95% CI: 3.21–4.69), and physical activity was positively associated with screening (AOR: 1.43, 95% CI: 1.11–1.82). (4) Conclusions: CRC screening uptake in Riyadh is critically low, highlighting the need for targeted public health interventions. Key predictors such as age, insurance coverage, smoking, and physical activity underscore the importance of addressing sociodemographic disparities and promoting health awareness. The findings emphasize the need for culturally tailored educational campaigns, improved healthcare access, and enhanced screening programs to increase uptake.

## 1. Introduction

Colorectal cancer (CRC) is a major global health issue, ranking as the third most common cancer and the second leading cause of cancer-related deaths worldwide [1,2]. Organized screening programs have demonstrated effects on cancer incidence and mortality [3]. In Saudi Arabia, CRC holds a significant position in the cancer landscape, being the most common cancer among men and the third most common among women [4,5]. The incidence of CRC in Saudi Arabia is rising, potentially due to lifestyle changes, an aging population, and increased adoption of Western dietary habits [6].

The development of CRC is a gradual process, typically evolving from benign adenomatous polyps to malignant tumors over several years [7]. Early-stage CRC often presents without noticeable symptoms, with indicators like rectal bleeding and changes in bowel habits usually appearing in later stages, which significantly diminish the chances of survival [8,9]. Early detection is crucial, as localized CRC has a five-year survival rate of 90%, contrasting sharply with the 10% survival rate for patients with metastatic disease [10,11]. Globally, standard screening guidelines recommend initiating CRC screening for average-risk individuals between 45 and 49 years of age [12]. Individuals with a higher risk, such as those with a family history of CRC or genetic conditions like Lynch syndrome, are advised to begin screening earlier and more frequently [11]. These guidelines underscore the importance of early intervention in reducing the burden of CRC [11].

In Saudi Arabia, CRC is often diagnosed at a younger average age of 60 compared to Western countries [13]. The increasing occurrence in individuals over 50, along with the prevalence of risk factors such as obesity, smoking, diabetes, and inflammatory bowel disease, highlights the necessity for a tailored screening strategy [14,15]. Furthermore, comorbidities like diabetes and hypertension have been shown to significantly increase the risk of CRC. Hyperinsulinemia and chronic inflammation associated with diabetes, as well as hypertension potentially leading to oxidative stress and gut microbiome alterations, may contribute to or accelerate CRC progression [16,17,18]. This emphasizes the importance of incorporating regular screening for high-risk individuals into comprehensive healthcare plans [19,20]. Effective screening programs not only improve survival outcomes but also lessen the economic strain associated with advanced stages of the disease [21]. Efforts to inform the Saudi population about the risks and consequences of CRC are likely carried out through public health campaigns, educational materials distributed in healthcare settings, and awareness initiatives by healthcare professionals.

Addressing CRC in Saudi Arabia involves tackling specific cultural and systemic factors [22]. Public health strategies should prioritize individuals with genetic predispositions, a history of polyps, or lifestyle risk factors like smoking and high-fat diets [23]. To enhance screening participation, it is vital to conduct awareness campaigns that address misconceptions about screening procedures and emphasize their benefits [24]. Factors such as higher education and income levels, along with systemic factors like physician referrals, have been observed to influence CRC screening uptake [25]. Commonly reported challenges include fear of diagnosis, limited awareness about screening, geographical disparities in access to healthcare, and systemic inadequacies [26,27,28]. Additionally, individual beliefs about their susceptibility to the disease and their confidence in the effectiveness of screening play a significant role in their decision to participate [29]. Understanding the various factors that influence CRC screening behaviour among the Saudi population is crucial for developing effective public health interventions. This study aims to investigate the demographic, behavioural, and systemic determinants of screening participation, with a particular focus on the role of comorbidities such as diabetes and hypertension. By providing evidence-based insights, the findings are intended to guide the development of culturally and institutionally appropriate interventions in Saudi Arabia, ultimately leading to improved screening rates and better cancer outcomes.

## 2. Materials and Methods

### 2.1. Study Design, Duration, and Setting

This study employed a cross-sectional survey design and was conducted from March 2023 to July 2023. It was conducted within the context of the Saudi Arabian Ministry of Health’s (MOH) Health Sector Transformation Program, a key initiative of Saudi Vision 2030. This program, initiated between 2021 and 2022, aimed to restructure the healthcare system from a traditional, centralized model to a health cluster model. This transition involved decentralizing healthcare delivery, integrating services across different levels of care, and enhancing efficiency and patient-centeredness. The study was conducted in the Riyadh region, which comprises three health clusters managed by Central Health Services. These clusters include primary healthcare centers (PHCs) and hospitals. Out of the 105 PHCs in the region, 48 were selected for participation in this study.

### 2.2. Sampling Technique

A multi-stage cluster sampling method was used to ensure a representative sample of individuals utilizing PHCs in the Riyadh region. The sampling process involved several steps. Riyadh was initially divided into three health clusters, with Health Cluster 2 selected for the study due to its diverse population and extensive healthcare infrastructure, serving approximately 3.7 million residents and encompassing 105 PHCs. From Health Cluster 2, 48 PHCs were randomly chosen using a stratified random sampling method to ensure proportional representation across urban and suburban areas. Within each of the selected PHCs, eligible participants were identified through systematic random sampling. This methodology ensured a representative sample of the general population utilizing primary healthcare services in Riyadh while minimizing potential selection bias.

### 2.3. Eligibility Criteria and Study Sample

The study targeted adults aged 18 and above who visited the selected PHCs, including both Saudi and non-Saudi residents. Individuals accessing primary healthcare services, regardless of their residency status, were eligible to participate. However, the study excluded healthcare practitioners and staff working at PHCs, individuals under the age of 18, patients with cognitive impairments that hindered their ability to understand the survey, and individuals who refused to provide informed consent. A total of 14,239 participants completed the electronic survey, which served as the final sample size for the analysis.

### 2.4. Development and Description of the Study Questionnaire

The study questionnaire was developed through a collaborative effort between the Central Health Services Reform Management Team and consultants representing various regions of Saudi Arabia. As a component of the broader health system reform initiative, a standardized tool was created to evaluate health perceptions, behaviors, and priorities across all health clusters nationwide. The questionnaire was structured into multiple sections, addressing key areas such as self-reported health status, health priorities and concerns, and health-related behaviors, including smoking, fast food consumption, physical activity, and alcohol use. Additionally, it assessed health perception on a scale from excellent to poor, collected sociodemographic data (e.g., age, education level, employment status, sex, and marital status, which was collected as a binary variable, with participants categorized as either “married” or “single”), and gathered information on medical history and comorbidities, such as heart disease, diabetes, obesity, hypertension, and hypercholesterolemia. Insurance coverage details were also included and study participants were asked about whether they have ever undergone any recommended colorectal cancer screening, regardless of type or method. This comprehensive design ensured that the questionnaire captured a diverse array of factors influencing health outcomes and healthcare utilization patterns within the population.

### 2.5. Validation and Reliability Assessment of the Questionnaire

To ensure the questionnaire’s validity and reliability, a rigorous evaluation process was implemented. Content validity was established by a panel of 15 experts, comprising healthcare professionals and public health specialists, who reviewed the questionnaire for relevance, clarity, and appropriateness. Based on their feedback, certain items were modified or removed to enhance the tool’s effectiveness. Face validity was assessed through a pilot study involving 200 participants, who evaluated the clarity, difficulty, and comprehensibility of the questions. To further confirm understanding, trained data collectors read the questions aloud during interviews. Reliability was tested using a test–retest approach, where 100 participants from the pilot study completed the questionnaire a second time via phone after initial adjustments were made. The test–retest reliability coefficient was calculated at 0.83, demonstrating high consistency and reliability. Additionally, to ensure linguistic precision, the questionnaire was translated from English to Arabic and then back translated to English, maintaining the accuracy and integrity of the tool across both languages.

### 2.6. Pilot Study and Rationale for Selecting Hail City

The pilot study was carried out in Hail City instead of Riyadh, as the Central Health Services Reform Management Team identified Hail as an ideal testing site. This choice was driven by Hail’s demographic and health profile, which closely mirrors that of the broader Saudi population, making it a suitable representative location for preliminary testing. The pilot study engaged 100 patients and 20 focus group participants, who were tasked with evaluating the clarity, comprehensibility, and difficulty of the questionnaire items. Their input provided critical insights, leading to necessary revisions and refinements to the questionnaire. These modifications ensured the tool’s effectiveness before its large-scale implementation in Riyadh and other health clusters across Saudi Arabia.

### 2.7. Data Collection Process

Data collection was conducted using an electronic survey administered by trained data collectors. The survey was designed and programmed onto iPads or Android tablets for use at the participating PHCs. Data collectors verified participant eligibility before inviting them to participate, ensuring only individuals aged 18 and above were included. The survey was administered through an interview-based approach, with data collectors reading questions aloud and recording responses on the tablets. The survey collected self-reported data from participants, including their history of CRC screening. It did not directly link to patient medical records or tumor characteristics. Instead, it focused on identifying predictors of self-reported CRC screening behaviour based on sociodemographic, behavioural, and health-related factors. This method ensured accuracy, minimized missing data, and maintained efficiency. Participants provided information on sociodemographic characteristics (e.g., age, gender, household size, marital status, education level, employment status, and health status), behavioural factors (e.g., smoking, fast food consumption, alcohol use, and physical activity), and comorbidities (e.g., hypertension, diabetes, obesity, and COPD). Participants also provided data on whether they had undergone any recommended colorectal cancer screening method in their lives, regardless of type or method. Participation in the survey was entirely voluntary, and informed consent was obtained from all participants before data collection.

### 2.8. Statistical Analysis

The statistical analysis began with an examination of the distribution of variables using histograms and p-p plots to assess normality. Continuous variables that followed a normal distribution, such as age, were summarized using means and standard deviations. Later, the age variable was categorized into groups to evaluate the distribution of participants across different age ranges. For categorical variables, including education level, employment status, marital status, health status, and insurance coverage, frequencies and proportions were calculated to describe the sample characteristics. Univariate analysis was performed using logistic regression to identify potential predictors associated with the outcome of interest, which was binary (colorectal cancer screening: Yes/No). Variables with a *p*-value of 0.25 or lower in the univariate analysis were considered eligible for inclusion in the multivariable logistic regression model. This threshold was chosen to ensure that all potentially relevant factors were retained for further analysis while minimizing the risk of excluding significant predictors.

In the multivariable logistic regression analysis, variables were systematically evaluated to identify characteristics that significantly predicted the outcome. Adjusted odds ratios (AOR) and their corresponding 95% confidence intervals (CI) were estimated and reported to quantify the strength and direction of associations. A *p*-value of less than 0.05 was used as the criterion for statistical significance. This approach allowed for the identification of independent predictors while controlling for potential confounding factors. All statistical analyses were conducted using SPSS version 26.0 (Statistical Package for Social Sciences) software, ensuring robust results. The selection of variables for both univariate and multivariable analyses was guided by their clinical relevance, distribution patterns, and preliminary associations observed in the data. This systematic approach ensured a comprehensive evaluation of factors influencing health perception and other outcomes of interest.

## 3. Results

### 3.1. Sociodemographic Characteristics of Study Participants

The study analyzed the sociodemographic characteristics, health status, and behavioural factors of 14,239 participants as shown in Table 1. On average, participants were 59.8 ± 16.35 years old. Most participants were aged 50–75 years (48.8%), followed by those under 50 years (34.0%) and at least 75 years (17.2%). A majority of participants were married (65.3%) and female (56.6%). Regarding education, more than half (51.5%) had completed college or university, while a smaller proportion reported primary education (4.0%). The employment status of the participants showed a near-equal distribution, with 51.4% employed and 48.6% unemployed as illustrated in Table 1.

In terms of health, 69.3% of participants rated their health as “excellent” or “very good”, while only 2.1% reported “poor” health. However, a striking 75.7% lacked insurance coverage. Behavioural analysis showed that most participants were physically active (60.7%) and non-smokers (72.3%). The prevalence of chronic conditions, including hypertension (11.1%), obesity (5.2%), and diabetes (12.4%), was relatively low (Table 1). Despite these indicators, colorectal cancer screening was alarmingly low, with only 4.2% of participants undergoing screening. These findings highlight key demographic and health-related trends, emphasizing the need for improved health insurance coverage and enhanced cancer screening programs.

### 3.2. Sociodemographic Predictors for Colorectal Cancer Screening

Table 2 illustrates sociodemographic predictors for colorectal cancer screening among Saudis at primary healthcare settings in Riyadh. The multivariate analysis identified key sociodemographic factors influencing colorectal cancer screening uptake among participants in primary healthcare settings in Riyadh. Age was a significant predictor, with participants aged 50–75 years being almost twice as likely to undergo screening compared to those under 50 years (AOR: 1.90, 95% CI: 1.50–2.42). Similarly, individuals aged 75 years or older showed higher odds of screening (AOR: 1.37, 95% CI: 1.01–1.87), emphasizing the need to prioritize older populations for screening initiatives. Education levels also played a role, as participants with “other” educational backgrounds were less likely to be screened compared to those with primary education (AOR: 0.60, 95% CI: 0.38–0.95). However, no significant associations were observed for those with high school or college/university education.

Gender and marital status did not show significant influence in the adjusted analysis. Males and females had comparable screening rates (AOR: 1.04, 95% CI: 0.87–1.25), and marital status was not a significant factor (AOR: 1.21, 95% CI: 0.97–1.52). In contrast, employment status showed a surprising trend, with unemployed participants being more likely to undergo screening than employed individuals (AOR: 1.85, 95% CI: 1.53–2.23). This suggests that unemployment may provide individuals with more time to access healthcare or that public health programs effectively target this group. Insurance coverage was another strong predictor, with insured participants being significantly more likely to be screened than their uninsured counterparts (AOR: 1.64, 95% CI: 1.37–1.96), underscoring the critical role of health insurance in promoting preventive care.

These findings highlight the importance of targeting older adults, uninsured populations, and individuals with non-traditional education backgrounds in colorectal cancer screening programs. Additionally, strategies should ensure that unemployed individuals continue to benefit from accessible screening services while addressing systemic barriers for those without insurance.

### 3.3. Behavioural and Health-Related Predictors Colorectal Cancer Screening

Table 3 illustrates the multivariate analysis which highlights several significant behavioural and health-related predictors of colorectal cancer screening among participants in primary healthcare settings in Riyadh.

The multivariate analysis revealed several significant behavioural and health-related predictors of colorectal cancer screening among participants in Riyadh. Smoking was strongly associated with screening uptake, as smokers were nearly four times more likely to undergo screening than non-smokers (AOR: 3.87, 95% CI: 3.21–4.69), suggesting that smoking-related health awareness or targeted interventions may play a role. Similarly, physical activity emerged as a significant predictor, with physically active individuals being more likely to participate in screening compared to inactive individuals (AOR: 1.43, 95% CI: 1.11–1.82). This finding highlights the link between healthier lifestyle behaviors and preventive healthcare engagement (Table 3).

While fast food consumption initially appeared associated with higher screening rates in simpler models, it was not significant in the fully adjusted analysis (AOR: 1.27, 95% CI: 0.97–1.68), indicating potential confounding factors. Similarly, diabetes showed a borderline association with screening (AOR: 1.29, 95% CI: 0.99–1.69), though the result was not statistically significant, emphasizing the need for more inclusive efforts to target individuals with chronic conditions. In contrast, hypertension, hypercholesterolemia, and heart disease were not significant predictors in the adjusted model, suggesting that these conditions alone do not influence screening behaviour (Table 3).

These findings underscore the importance of targeting smokers and physically active individuals in colorectal cancer screening campaigns. Additionally, there is a need to address barriers faced by less active individuals and ensure that those with chronic conditions like diabetes are included in screening programs. Efforts should focus on promoting broader awareness of screening benefits across all demographic and health groups.

## 4. Discussion

The findings of this study highlight alarmingly low CRC screening rates among participants in Riyadh’s primary healthcare settings, with only 4.2% of individuals undergoing screening. This figure is significantly lower than the rates observed in developed countries, such as the United States, where screening participation can exceed 60% [30], and certain European nations, where organized screening programs have increased participation to over 22 to 66% depending upon the type of screening method [31]. These differences underline the need for context-specific strategies to address barriers to screening in Saudi Arabia.

The association between older age and higher CRC screening uptake aligns with global trends, as screening guidelines primarily target individuals aged 50–75 years due to their heightened risk [12,32]. However, the relatively modest increase in screening among older participants in this study points to gaps in both awareness and access to preventive care, even within this priority age group. Interestingly, individuals aged 75 years and older also had increased odds of screening. This finding is somewhat unexpected, as many international guidelines recommend discontinuing routine CRC screening at advanced ages unless specific risk factors are present [32]. It is possible that differences in physician recommendations or patient behaviors in Saudi Arabia contribute to this pattern. However, future studies can discover the reasons for high odds of screening in the older age group.

Our study found a high proportion (75%) of participants without health insurance. This finding is likely related to the study population, which included both Saudi citizens and non-Saudi residents. In Saudi Arabia, healthcare is provided through a mixed system of public and private sectors. The government provides free healthcare to Saudi citizens through a network of public hospitals and primary healthcare centers. However, expatriates and some private-sector employees typically rely on private health insurance. The prevalence of health insurance coverage can vary depending on employment sector, nationality, and socioeconomic status. While Saudi citizens have access to free government-provided healthcare, non-Saudi residents typically require private health insurance, often tied to employment. Factors such as employment in sectors with limited benefits, temporary residency status, or unemployment can contribute to lack of insurance among this group. Additionally, while not fully explored in our study, socioeconomic factors may play a role in insurance coverage even among citizens. The high percentage of uninsured in our study highlights a potential barrier to healthcare access, including CRC screening, for a significant portion of the population. This is consistent with other studies that have identified gaps in insurance coverage as a barrier to healthcare access in the region [33,34,35]. In the current study, health insurance emerged as a significant predictor of screening, reflecting its pivotal role in facilitating access to preventive services. This finding is consistent with evidence from other countries, which demonstrates that individuals with health insurance are more likely to participate in preventive care, including CRC screening [33,34,35]. In Saudi Arabia, the observed disparity highlights the systemic inequities faced by uninsured populations, who may encounter financial and logistical barriers to accessing these services. Beyond individual insurance status, system-level barriers such as the availability of colonoscopy services, the capacity of primary healthcare centers to offer or refer for fecal occult blood tests or other screening modalities, and the efficiency of referral pathways likely contribute to the low screening rates [22,36]. Research from Saudi Arabia has highlighted challenges in the implementation of national screening programs, including infrastructure limitations and resource allocation [22,36].

An unexpected observation was the strong association between smoking and CRC screening uptake. Smokers in this study were nearly four times more likely to undergo screening compared to non-smokers. While this contrasts with findings from some Western studies where smokers tend to avoid preventive care [37,38,39], it may be explained by heightened health awareness among smokers in Saudi Arabia due to anti-smoking campaigns. Alternatively, this association could reflect the higher prevalence of comorbidities among smokers, prompting physicians to recommend screening as part of their clinical management. Indeed, prior research consistently demonstrates the significant effect of physician recommendation on patient adherence to colorectal cancer screening [40,41]. Healthcare providers are key in educating patients and promoting screening, particularly for high-risk groups like smokers. However, future large quantitative and qualitative studies are needed to understand the causes and potential explanations for such findings.

Physical activity was another significant predictor in the current analysis, with active individuals showing greater screening uptake than their inactive counterparts. This aligns with findings from other studies suggesting that healthier lifestyle behaviors are often linked to better adherence to preventive healthcare measures [25,42]. Those who engage in regular physical activity may have higher health awareness and a greater likelihood of interacting with healthcare systems, thereby increasing their opportunities for screening [43].

Despite these insights, the low overall screening rate underscores systemic and cultural barriers to CRC screening in Saudi Arabia. Fear of invasive procedures, lack of awareness about screening benefits, misconceptions about CRC prevention, and limited access to health care providers are likely contributors [44,45], though these need to be further explored in future studies. Comparing our findings with other Middle Eastern settings reveals a similarly low awareness of CRC symptoms and risk factors. A study in Qatar among adults aged 50–74 years attending primary healthcare centers found a low mean awareness score, with men, Qataris, and those with no formal education having the lowest awareness [46]. Furthermore, research among Arab Americans in Michigan, USA, indicated that adherence to CRC screening varied significantly by country of origin, with Yemenis having much lower screening rates compared to Lebanese, despite similar socioeconomic factors. Lack of awareness about screening was a major barrier for unscreened Yemenis [47]. In Palestine, a national study identified cultural and religious barriers, lack of education, unfamiliarity with screening, distrust of Western medicine, and embarrassment as factors associated with decreased willingness to undergo CRC screening [48]. These findings from Qatar, the USA, and Palestine highlight the diverse challenges and the need for culturally tailored interventions to improve CRC screening uptake in different populations within and beyond the Middle East. Further research in Saudi Arabia should explore the role of educational level and gender in CRC screening uptake.

This study has several key strengths that enhance the validity and applicability of its findings. First, the large sample size and use of multistage random sampling ensure a representative and diverse participant pool, improving the generalizability of the results to similar populations in Saudi Arabia and beyond. The inclusion of both insured and uninsured individuals provides a comprehensive understanding of healthcare access disparities, offering valuable insights into the factors influencing CRC screening uptake. Additionally, the study employed rigorous multivariate analysis, which allowed for the identification of significant predictors while controlling for potential confounding variables. The use of a validated and reliable questionnaire further strengthened the study by minimizing measurement errors and ensuring the accuracy of the collected data.

Despite these strengths, our study has some limitations that must be acknowledged. The reliance on self-reported data introduces the potential for recall bias and social desirability bias, particularly for sensitive behaviors such as smoking, physical activity, and screening status. These biases may affect the accuracy of the reported data and the validity of the findings. The study’s reliance on self-reported data for several key parameters presents a further limitation. Specifically, the questionnaire did not differentiate between current and past smokers, nor did it specify the frequency or intensity of physical activity. This lack of detailed information limits our ability to draw precise conclusions about the relationship between these factors and CRC screening uptake. Furthermore, the cross-sectional design of the study limits our ability to establish causal relationships between the predictors and CRC screening uptake. Longitudinal studies would be necessary to explore these causal links more definitively. While the study provides important insights, its focus on the Riyadh region may not fully capture regional variations in screening practices and healthcare access across Saudi Arabia. This limits the generalizability of the findings to other areas of the country, where cultural, economic, and healthcare infrastructure differences may influence screening behaviors. Furthermore, this study did not correlate factors that examined tumor characteristics, such as the stage of cancer at diagnosis. Future research could explore these associations to provide a more comprehensive understanding of CRC outcomes. Additionally, the study did not specify the exact types of CRC screening methods used by participants, which limits our ability to draw conclusions about the effectiveness of specific screening modalities. However, we have made it clear that the study asked about screening regardless of type or method. Lastly, the study categorized marital status into only two groups, “married” and “single,” which limits our ability to examine the influence of other marital statuses, such as divorced or widowed, on CRC screening uptake.

## 5. Conclusions

This study reveals a critically low CRC screening rate in Riyadh. Age, health insurance, smoking, and physical activity significantly influence screening uptake. Targeted public health strategies are urgently needed to address barriers, particularly among high-risk and underserved groups. Multi-faceted interventions should include culturally sensitive education, expanded insurance coverage, enhanced primary care provider involvement, community-based health promotion, and improved access to screening. These efforts are essential to increase screening rates and improve cancer outcomes in the region.

## Figures and Tables

**Table 1:** Sociodemographic characteristics of study participants who completed the survey (n = 14,239).

Age	Frequency	Percentage
<50 years	4848	34
50 to 75 years	6945	48.8
At least 75 years	2446	17.2
Gender		
Female	8062	56.6
Male	6177	43.4
Marital status		
Single	4939	34.7
Married	9300	65.3
Education		
Primary	572	4.0
Up to high school	3937	27.6
College/university	7336	51.5
Others	2394	16.8
Employment status		
Employed	7317	51.4
Unemployed	6922	48.6
Health status		
Excellent	4798	33.7
Very good	5076	35.6
Good	2815	19.8
Fair	1256	8.8
Poor	294	2.1
Insurance coverage		
No	10,782	75.7
Yes	3457	24.3
Physical activity		
No	5598	39.3
Yes	8641	60.7
Smoking		
No	10,297	72.3
Yes	3942	27.7
Hypertension		
No	12,659	88.9
Yes	1580	11.1
Obesity		
No	13,502	94.8
Yes	737	5.2
Diabetes		
No	12,474	87.6
Yes	1765	12.4
Screening for Colorectal Cancer		
No	13,644	95.8
Yes	595	4.2

**Table 2:** Sociodemographic predictors for colorectal cancer screening among Saudis at primary healthcare settings in Riyadh.

	Findings of Univariate Analysis			Findings of Multivariable Analysis		
Predictors		95% CI			95% CI	
Age	OR	LL	UL	AOR	LL	UL
<50 years	1.00			1.00		
50 to 75 years	1.87	1.53	2.28	1.90	1.50	2.42
At least 75 years	1.47	1.13	1.91	1.37	1.01	1.87
Education						
Primary	1.00			1.00		
Up to high school	0.78	0.53	1.20	0.91	0.60	1.38
College/university	0.96	0.650	1.43	1.14	0.76	1.71
Others	0.54	0.34	0.85	0.60	0.38	0.95
Gender						
Female	1.00			1.00		
Male	0.87	0.74	1.03	1.04	0.87	1.25
Marital status						
Single	1.00					
Married	1.53	1.27	1.85	1.21	0.97	1.52
Employment status						
Employed	1.00			1.00		
Unemployed	1.36	1.15	1.61	1.85	1.53	2.23
Insurance coverage						
No	1.00			1.00		
Yes	1.59	1.33	1.89	1.64	1.37	1.96

OR: Odds ratio; AOR: Adjusted odds ratio; LL: Lower limit, UL: Upper limit; 95% CI: 95% confidence intervals.

**Table 3:** Behavioural and health-related predictors for colorectal cancer screening among Saudis at primary healthcare settings in Riyadh.

Predictors	Univariable Analysis			Adjusted for Age and Sex			Multivariable Analysis		
		95% CI			95% CI			95% CI	
Smoking	OR	LL	UL	AOR	LL	UL	AOR	LL	UL
No	1.00						1.00		
Yes	4.64	3.91	5.50	4.75	4.00	5.64	3.87	3.21	4.69
Physical activity									
No	1.00			1.00			1.00		
Yes	2.77	2.26	3.41	2.76	2.25	3.39	1.43	1.11	1.82
Fast food consumption									
No	1.00			1.00			1.00		
Yes	2.42	1.91	3.07	2.45	1.93	3.12	1.27	0.97	1.68
Diabetes									
No	1.00			1.00			1.00		
Yes	1.45	1.16	1.81	1.40	1.12	1.76	1.29	0.99	1.69
Hypertension									
No	1.00			1.00			1.00		
Yes	1.56	1.24	1.95	1.50	1.19	1.89	1.15	0.85	1.55
Hypercholesterolemia									
No	1.00			1.00			1.00		
Yes	1.83	1.46	2.30	1.74	1.39	2.20	1.17	0.87	1.57
Heart disease									
No	1.00			1.00			1.00		
Yes	2.41	1.79	3.24	2.37	1.76	3.19	1.16	0.82	1.65

OR: Odds ratio; AOR: Adjusted odds ratio; LL: Lower limit, UL: Upper limit; 95% CI: 95% confidence intervals.

## Data Availability

Data is contained within the article.

## Abbreviations

The following abbreviations are used in this manuscript:

MDPI	Multidisciplinary Digital Publishing Institute
DOAJ	Directory of open access journals
CRC	Colorectal cancer
OR	Odds ratio
CI	Confidence interval
AOR	Adjusted odds ratio
LL	Lower limit
UL	Upper limit
